# Intracellular osmoprotectant concentrations determine *Propionibacterium freudenreichii* survival during drying

**DOI:** 10.1007/s00253-020-10425-1

**Published:** 2020-02-19

**Authors:** Floriane Gaucher, Houem Rabah, Koffigan Kponouglo, Sylvie Bonnassie, Sandrine Pottier, Anne Dolivet, Pierre Marchand, Romain Jeantet, Philippe Blanc, Gwénaël Jan

**Affiliations:** 1 UMR STLO, INRAE, Agrocampus Ouest, 35042 Rennes, France; 2 Bioprox, 6 rue Barbès, 92532 Levallois-Perret, France; 3 Pôle Agronomique Ouest, Régions Bretagne et Pays de la Loire, 35042 Rennes, France; 4 Université de Rennes I, Rennes, France; 5 CNRS, ISCR - UMR 6226, University Rennes, PRISM, BIOSIT – UMS 3480, 35000 Rennes, France

**Keywords:** Osmoadaptation, Spray-drying, Propionibacteria, Viability, Stress, Cross-protection

## Abstract

*Propionibacterium freudenreichii* is a beneficial bacterium widely used in food as a probiotic and as a cheese-ripening starter. In these different applications, it is produced, dried, and stored before being used. Both freeze-drying and spray-drying were considered for this purpose. Freeze-drying is a discontinuous process that is energy-consuming but that allows high cell survival. Spray-drying is a continuous process that is more energy-efficient but that can lead to massive bacterial death related to heat, osmotic, and oxidative stresses. We have shown that *P. freudenreichii* cultivated in hyperconcentrated rich media can be spray-dried with limited bacterial death. However, the general stress tolerance conferred by this hyperosmotic constraint remained a black box. In this study, we modulated *P. freudenreichii* growth conditions and monitored both osmoprotectant accumulation and stress tolerance acquisition. Changing the ratio between the carbohydrates provided and non-protein nitrogen during growth under osmotic constraint modulated osmoprotectant accumulation. This, in turn, was correlated with *P. freudenreichii* tolerance towards different stresses, on the one hand, and towards freeze-drying and spray-drying, on the other. Surprisingly, trehalose accumulation correlated with spray-drying survival and glycine betaine accumulation with freeze-drying. This first report showing the ability to modulate the trehalose/GB ratio in osmoprotectants accumulated by a probiotic bacterium opens new perspectives for the optimization of probiotics production.

## Introduction

*Propionibacterium freudenreichii* is a generally-recognized-as-safe (GRAS), food-grade, beneficial bacterium with QPS status (EFSA experts 2008). It is used as a multi-purpose starter in the dairy and probiotic industry (Thierry et al. 2011). Its technological applications include Swiss-cheese manufacturing and production of antimicrobial molecules and of nutritional molecules (Rabah et al. 2017). Its obligatory fermentative metabolism leads to the production of the beneficial short-chain fatty acids, acetate, and propionate, as final products, concomitantly with the release of vitamins B9 (folate) and B12 (cobalamin) and of the bifidogenic compounds, DNA (1,4-dihydroxy-2-naphtoic acid) and ACNQ (2-amino-3-carboxy-1,4-naphthoquinone) (Rabah et al. 2017). This led *P. freudenreichii* to be described as a nutraceutical producer (Hugenholtz et al. 2002). Consumption of selected strains of *P. freudenreichii* modulates the gut microbiota in the context of colitis as well as in healthy conditions (D. Bouglé et al. 1999; Hojo et al. 2002; Seki et al. 2004; Mitsuyama et al. 2007). This microbiota modulation, in favor of symbiont bifidobacteria and at the expense of pathobiont Bacteroides, depends on the release of the bifidogenic small molecules, DHNA and ACNQ, by propionibacteria (Isawa et al. 2002). *P. freudenreichii* modulates the mucosal immune response. It was shown to induce production of anti-inflammatory cytokines in immune (Foligne et al. 2010) and epithelial (Rabah et al. 2018) human cells, although the intensity of this induction varies with the strain studied (Foligné et al. 2013). Accordingly, consumption of *P. freudenreichii* protected animals from inflammatory intestinal disease induced by TNBS (2,4,6-trinitrobenzenesulfonic acid) (Foligne et al. 2010; Plé et al. 2015, 2016), by DSS (Dextran Sodium Sulfate) (Okada et al. 2006b), by NSAID (Nonsteroidal anti-inflammatory drugs) (Okada et al. 2006a), and by 5-FU (5-fluorouracil) (Cordeiro et al. 2018). Pilot studies also reported a healing effect in the context of ulcerative colitis in humans (Mitsuyama et al. 2007; Okada et al. 2006b). The release of propionibacterial metabolites in contact with intestinal epithelial cells was further shown to favor apoptotic depletion of cancer cells in vitro (Jan et al. 2002; Lan et al. 2007; Cousin et al. 2016) and in animals (Lan et al. 2008), which may enhance the efficacy of available treatments aimed at preventing or treating digestive cancers (Cousin et al. 2016). To trigger such beneficial effects in the digestive tract, it is crucial for propionibacteria to be consumed alive, which closely depends on the drying technology used.

We have recently shown that although normal laboratory cultures of *P. freudenreichii* experience massive cell death upon spray-drying, growth in hyperconcentrated sweet whey dramatically enhances its survival during this stressful process. Sweet whey constitutes a very rich and complex growth medium that was used in a four- to five-time concentrated hypertonic form in this study. Growth of *P. freudenreichii* in such medium triggered complex and diverse modifications within propionibacteria including changes in morphology; overexpression of stress proteins including ClpB, SodM, EF-Tu, and Hsp20; and accumulation of storage compounds including glycogen and polyphosphate (Huang et al. 2016c). During osmoadaptation, *P. freudenreichii* is able to accumulate trehalose, glycine betaine, and glutamate (Gaucher et al. 2019). Trehalose accumulation has already been reported to protect bacteria during freeze-drying (Termont et al. 2006) and glycine betaine accumulation can have a positive or negative impact on drying depending on the bacterial species (Saarela et al. 2005; Sheehan et al. 2006; Bergenholtz et al. 2012). However, tolerances conferred by each osmoprotectants are not well known. Owing to the great complexity of the hyperconcentrated sweet whey growth and drying medium, the mechanisms triggered that lead to general stress tolerance remained a black box. In particular, the nature and the amount of the different osmoprotectants accumulated were poorly addressed. Indeed, different osmoprotectants may be accumulated, with different impacts on tolerance acquisition.

In this study, we modulated *P. freudenreichii* growth conditions by changing concentrations of available carbohydrates (CH) and non-protein nitrogen (NPN). We monitored osmoprotectant accumulation and stress tolerance acquisition. Changing the ratio between CH and NPN during growth under osmotic constraint modulated the ratio between glycine betaine (GB) and trehalose. This, in turn, affected *P. freudenreichii* tolerance towards different stresses, towards freeze-drying, and towards spray-drying. This is, to our knowledge, the first report showing the ability to modulate the trehalose/GB ratio in osmoprotectants accumulated by a probiotic bacterium, in order to enhance drying efficacy. It opens new perspectives for the optimization of freeze-drying and spray-drying processes.

## Materials and methods

### Growth media and *P. freudenreichii* growth

*Propionibacterium freudenreichii* CIRM-BIA 129 (equivalent ITG P20) was provided, stored, and maintained by the CIRM-BIA Biological Resource Center (Centre International de Ressources Microbiennes-Bactéries d’Intérêt Alimentaire, INRA, Rennes, France). *P. freudenreichii* is routinely cultivated in yeast extract lactate (YEL) medium (Malik et al. 1968). *P. freudenreichii* CIRM-BIA 129 was grown in different media: laboratory medium—YEL medium, YEL medium with 0.9 M NaCl (YEL+NaCl), YEL medium with 34 g L^−1^ of lactose (YEL+lactose), and YEL medium with 34 g L^−1^ of lactose and 0.9 M NaCl (YEL+lactose+NaCl); and in dairy-type medium—sweet whey (SW), sweet whey with 0.7 M NaCl (SW+NaCl), milk ultrafiltrate (MU), and milk ultrafiltrate with 0.7 M NaCl (MU+NaCl). NPN was determined as previously described (Gripon et al. 1975) and total nitrogen (TN) was determined using the Ogg 1960). Osmolarity and growth medium composition are reported in Table 1. Bacterial populations were monitored by optical density (OD) at 650 nm. Salt concentrations are the highest concentrations that enable *P. freudenreichii* growth in the different media.

**Table 1:** Composition and osmotic pressures of the different culture media

	YEL	YEL+NaCl (0.9 M)	YEL+L	YEL+L+NaCl (0.9 M)	MU	MU+NaCl (0.7 M)	SW	SW+NaCl (0.7 M)
Saccharides (lactose) (g L^−1^)	0	0	34	34	48	48	33.4	33.4
Nitrogen total (g L^−1^)	14.6	14.6	14.6	14.6	2.3	2.3	0.5	4.2
Non-protein nitrogen (NPN) (g L^−1^)	14.2	14.2	14.2	14.2	1.5	1.5	0.2	1.6
Lactose/NPN	0	0	2.4	2.4	32	32	167	20.9
Osmotic pressure (osmol)	0.308	2.028	0.406	2.175	0.259	1.996	0.192	1.903

*YEL*: yeast extract lactate. *L*: lactose. *MU*: milk ultrafiltrate. *SW*: sweet whey

### Identification and quantification of osmoprotectants accumulated by *P. freudenreichii* CIRM-BIA 129

#### Extraction of accumulated osmoprotectants

*P. freudenreichii* CIRM-BIA 129 was grown in the different media. During the exponential phase (OD = 0.8), cells were harvested by centrifugation (8000*g*, 10 min, 30 °C). Osmoprotectants were then extracted as previously described (Gaucher et al. 2019). Cells were washed twice in a NaCl solution with the same osmolality as the culture medium. Cells were then re-suspended in 2 mL of distilled water, and 8 mL of ethanol absolute were then added. The suspension was homogenized and centrifuged (8000g, 10 min, 30 °C) in order to remove cell fragments. The supernatant extract was evaporated for 7 h with a rotary evaporator (Hei-VAP value Digital, Heidolph). Dried extracts were then solubilized in deuterium oxide (Sigma-Aldrich, USA).

#### Nuclear magnetic resonance analyses

Osmoprotectants were then identified and quantified by nuclear magnetic resonance (NMR) as previously described for other bacteria. All ^1^H and ^13^C NMR spectra were recorded at 298 K on a Bruker Avance 500 spectrometer equipped with a 5-mm TCI triple-resonance cryoprobe (PRISM Core Facility, Rennes). ^1^H spectra were acquired with a 6-kHz spectral width, 32 K data points, and a total repetition time of 6.73 s. ^13^C spectra were acquired using a proton power-gated decoupling sequence with a 30° flip angle, a 30-kHz spectral width, 64 K data points, and a total repetition time of 3.08 s. The data were processed with Topspin software (Bruker Biospin). Before applying the Fourier transform, free induction decays of ^1^H spectra were treated with an exponential broadening of 0.3 Hz.

Samples were solubilized in D_2_O (Sigma-Aldrich, USA). 3-(Trimethylsilyl)propionic-2,2,3,3-d4 acid sodium salt (TSP-d4) (Sigma-Aldrich, USA) served as an internal reference for ^1^H and ^13^C chemical shifts. The relative concentration of trehalose, glutamate, and glycine betaine in the samples was determined by the integration of the peaks areas of their ^1^H signals relative to the internal standard TMSP.

### Stress challenges

Heat, oxidative, bile salt, and acid challenges were applied to cultures at the beginning of the stationary phase (when maximal OD was reached). The heat challenge was performed by placing 2 mL (in a 15-mL Falcon tube) of *P. freudenreichii* culture in a water bath for 10 min at 60 °C, corresponding to the spray-drying temperature (Leverrier et al. 2004). The oxidative challenge was applied by adding 1.25 mM of hydrogen peroxide (Labogros, France) to 2 mL of *P. freudenreichii* culture for 1 h at 30 °C (Serata et al. 2016). The acid challenge was applied by re-suspending *P. freudenreichii* in culture medium adjusted to pH 2.0 by using HCl at 30 °C followed by a 1-h incubation (Jan et al. 2000). The bile salt challenge was performed by adding 1 g L^−1^ of a bile salt mixture (an equimolar mixture of cholate and desoxycholate; Sigma Chemical, St. Louis, MO, USA) to the culture for 1 h at 37 °C (Leverrier et al. 2003). CFU counting was performed after the challenge. In order to calculate the survival percentage, a CFU counting was made on untreated culture left for the same length of time at 30 °C (for the heat, oxidative, and acid challenges) or 37 °C (for the bile salt challenge) as a control.

### Spray-drying and powder storage

Spray-drying was performed on a laboratory-scale spray-dryer (Mobile Minor™, GEA Niro, Denmark). *P. freudenreichii* was cultivated in the different YEL media. At the beginning of the stationary phase, cells were harvested by centrifugation (8000*g*, 20 min, 30 °C) and re-suspended in a maltodextrin solution (DE = 6–8) (Roquette, France). In the bacterial solution obtained, maltodextrin concentration was 20% of the dry matter, whereas the bacterial concentration was 4% of the dry matter. These different suspensions (∼ 2 L) of *P. freudenreichii* were agitated for 10 min prior to delivery to the dryer by a peristaltic pump (520S, Watson-Marlow, France). A two-fluid nozzle with a diameter of 0.8 mm was used for atomization. The inlet air temperature was fixed at 160 °C. The temperature outlet air was controlled at 60 ± 2 °C, by adjusting the feed rate. The bacterial viabilities were estimated by numeration on YEL agar plates incubated at 30 °C before and after spray-drying, as previously described (Huang et al. 2016a). The powders were collected and sealed in sterilized polystyrene bottles (Gosselin, France); stored at a controlled temperature of 4, 20, or 37 °C; and kept away from light. The samples were stored for analyses for 112 days (approximately 4 months). One gram of powder was solubilized in 9 g of sterile water and bacterial viability was tested by numeration on YEL agar plates incubated at 30 °C.

### Freeze-drying

*P. freudenreichii* strains were grown in the YEL, YEL+NaCl, YEL+lactose, and YEL+lactose+NaCl media. At the beginning of the stationary phase, cultures were harvested (8000*g*, 10 min, 30 °C). Pellets were then homogenized in a maltodextrin solution (DE = 6–8) (Roquette, France) with a final concentration of 10% (*w*/*w*). The bacterial solutions were then freeze-dried (2253-04, Serail, France). The bacterial viabilities were estimated by numeration on YEL agar plates incubated at 30 °C before and after freeze-drying.

### Statistical analysis

Each analysis was done in 3 biological replicates. Hence, the results presented in this study are averages of three independent replicates or experiments. All the results are presented as a mean value with standard deviation. Statistical analyses were performed using one-way ANOVA with Tukey post hoc analyses for multiple comparison. Statistical significance was set at *p* < 0.05. Calculations were performed using GraphPad Prism Software (Prism 7 for Windows).

## Results

### *P. freudenreichii* growth parameters depend on carbon sources and on salt concentration

**Fig. 1:**
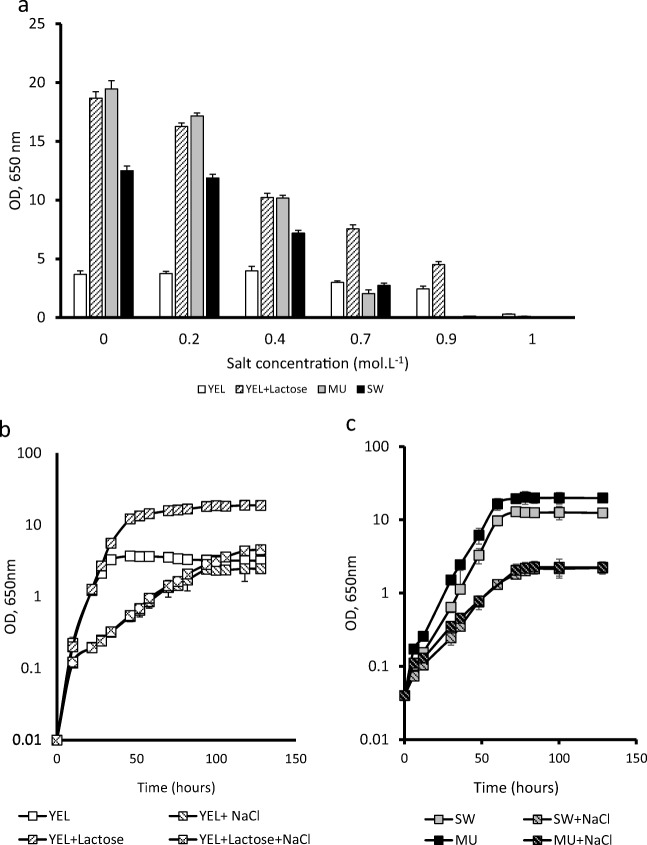
*P freudenreichii* growth in media supplemented or not with NaCl. **a** Final optical densities were measured after 130 h of growth in YEL-type media (YEL, YEL+NaCl YEL+lactose, and YEL+lactose+NaCl) and in dairy-type media (SW, SW+NaCl, MU and MU+NaCl) in different salt concentrations. **b**, **c***P. freudenreichii* CIRM-BIA 129 growth was then monitored in these media in the presence of the highest NaCl concentration that would allow growth. YEL: yeast extract lactate. MU: milk ultrafiltrate. SW: sweet whey

The addition of lactose (34 g L^−1^) to the rich YEL medium increased the final population of propionibacteria in agreement with *P. freudenreichii* ability to use both lactate and lactose, as shown in Fig. 1 a. MU (milk ultrafiltrate) and SW (sweet whey) both allowed growth of *P. freudenreichii* up to populations above those allowed by YEL. These media contain lactose. The presence of NaCl inhibited *P. freudenreichii* growth in all media. However, only the rich YEL medium sustained growth of *P. freudenreichii* up to 0.9 M NaCl, in agreement with the presence of osmoprotectants. The dairy culture media, MU (milk ultrafiltrate) and SW (sweet whey), failed to allow growth of *P. freudenreichii* in the presence of such high salt concentrations (Fig. 1a). The addition of salt (NaCl 0.9 M), to the rich YEL medium containing lactose, and NaCl 0.7 M to MU and to SW, decreased both parameters, i.e., growth rates and final populations, compared to a non-salty medium (Fig. 1b and c). In contrast, the addition of salt (NaCl 0.9 M) to YEL without lactose decreased the growth rate but the final population was maintained, compared to the YEL control. The addition of lactose to the YEL and to the YEL medium containing NaCl had no impact on the *P. freudenreichii* growth rate but increased final populations, in agreement with its role as a fermentation substrate. Accordingly, the pH decreased down to values close to 5 in all media containing lactose, while it remained close to 6.5 in YEL without lactose.

### Osmoprotectants accumulation in *P. freudenreichii* is medium-dependent

**Fig. 2:**
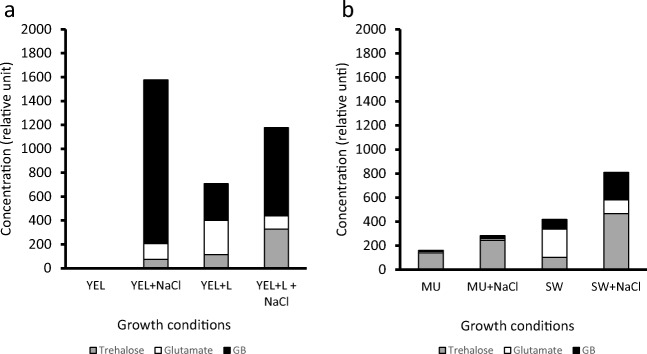
*P freudenreichii* osmoprotectants accumulation is medium-dependent. *P. freudenreichii* CIRM-BIA 129 was cultivated in YEL-type media (**a**) and dairy-type media (**b**) with or without NaCl. Cytoplasmic extracts were made, as described in the “Materials and methods” section. Intracellular osmoprotectants were then identified and quantified by NMR analysis. Osmoprotectants accumulations are expressed as relative units. YEL: yeast extract lactate. L: lactose. MU: milk ultrafiltrate. SW: sweet whey

Salt addition improved the total amount of osmoprotectants accumulated by *P. freudenreichii*, regardless of the culture medium (Fig. 2a and b). The presence of lactose had a similar impact: in YEL containing lactose and in YEL containing lactose and NaCl, the total amount of osmoprotectants accumulated was higher than that in YEL and in YEL containing NaCl, respectively. This is in agreement with the additional 0.1 osmol of osmotic pressure due to 34 g L^−1^ of lactose and with the acidification of the growth medium. As a negative control, the YEL medium, with an osmolality of 0.308 osmol, causes no osmotic stress and, consequently, no osmoprotectant accumulation inside bacteria. In the presence of salt (YEL containing NaCl), *P. freudenreichii* mainly accumulated glycine betaine (1370 relative units (RU)) and low amounts of glutamate (132 RU) and trehalose (74 RU). In the presence of lactose (YEL containing lactose and NaCl), *P. freudenreichii* accumulated higher proportions of trehalose (328 RU) and lower proportions of glycine betaine (739 RU). In MU and in SW, which are both rich in lactose, the addition of salt led *P. freudenreichii* to mainly accumulate trehalose (244 RU and 467 RU, respectively), as well as small amounts of glycine betaine and of glutamate (14 RU and 26 RU, respectively, for MU+NaCl and 228 RU, and 114 RU, respectively, for SW+NaCl).

In conclusion, the three growth media that contain lactose (YEL containing lactose, MU, and SW) triggered osmoprotectant accumulation even in the absence of salt. Salt triggered GB accumulation in YEL, and this accumulation was reverted by lactose addition, which leads to trehalose accumulation. In YEL containing NaCl, GB is the main compound accumulated, whereas in MU containing NaCl and SW containing NaCl, trehalose is the main compound accumulated.

### Multiple stress tolerance of *P. freudenreichii* is medium-dependent

**Fig. 3:**
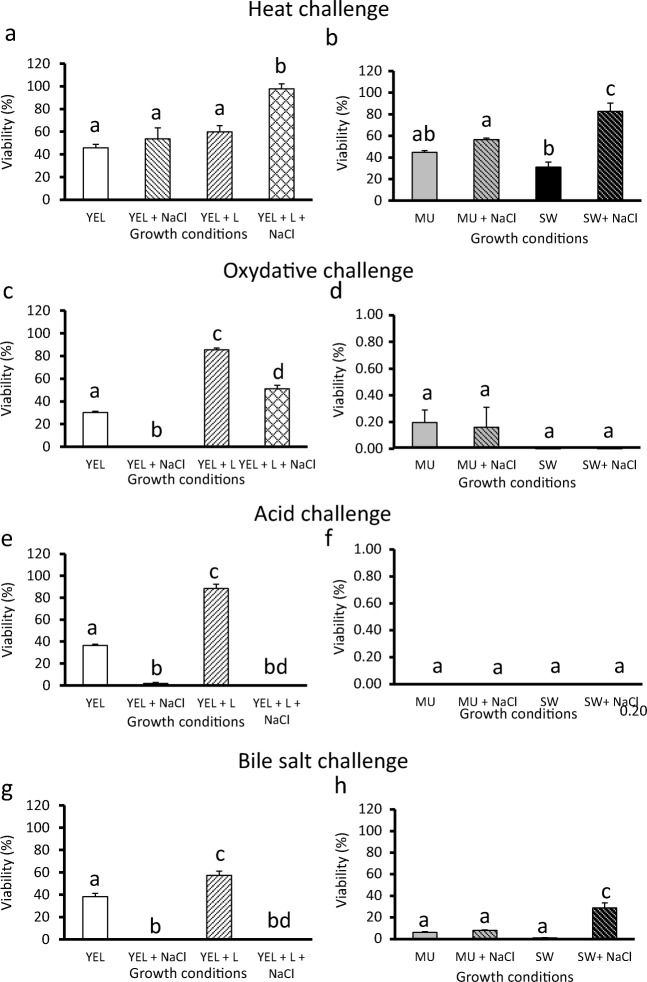
Cross-protections conferred to *P. freudenreichii* are medium-dependent. *P. freudenreichii* CIRM-BIA 129 was cultivated until the beginning of the stationary phase in YEL-type media (A, C, E, G) and dairy-type media (B, D, F, H), with and without NaCl. Cultures were then subjected to heat (A and B, 60 °C for 10 min), oxidative (C and D, 1.25 mM H_2_O_2_ for 1 h), acid challenges (E and F, pH = 2 for 1 h), or bile salts (G and H, 1 g L^−1^ for 1 h) challenges, as described in the “Materials and methods” section. *P. freudenreichii* viability was determined by CFU counting in treated and control samples. Results are expressed as a percentage of survival. Error bars represent the standard deviation for triplicate experiments. Significant differences are reported with different letters above the columns (*p* > 0.05). YEL: yeast extract lactate. L: lactose. MU: milk ultrafiltrate. SW: sweet whey

*P. freudenreichii* stress tolerance was monitored by subjecting bacteria to heat, oxidative, acid, and bile salt challenges. *P. freudenreichii* viability was determined by numeration before and after stress challenges. We selected a series of challenges relevant for both technological and digestive constraints. The presence of NaCl in YEL medium had no impact on *P. freudenreichii* heat resistance at 60 °C (Fig. 3(A)), but had a significant positive effect in the YEL containing lactose and NaCl, SW containing NaCl, and MU containing NaCl media (97.9%, 82.7%, and 56.6%, respectively), compared to the non-salty media (60.0%, 31.0%, and 44.8%, respectively) (Fig. 3(A, B)). However, the presence of salt in the four media (YEL containing NaCl, YEL containing lactose and NaCl, SW containing NaCl, and MU containing NaCl) had a significant negative effect or no impact on *P. freudenreichii* survival to oxidative, acid, and bile salt (Fig. 3(C–H)). These results reveal a cross-protection between osmoadaptation and heat stress, but not for the other stresses. The presence of lactose (YEL containing Lactose and YEL containing lactose and NaCl) had a significant positive impact for the four different lethal challenges.

### *P. freudenreichii* viability after drying

**Fig. 4:**
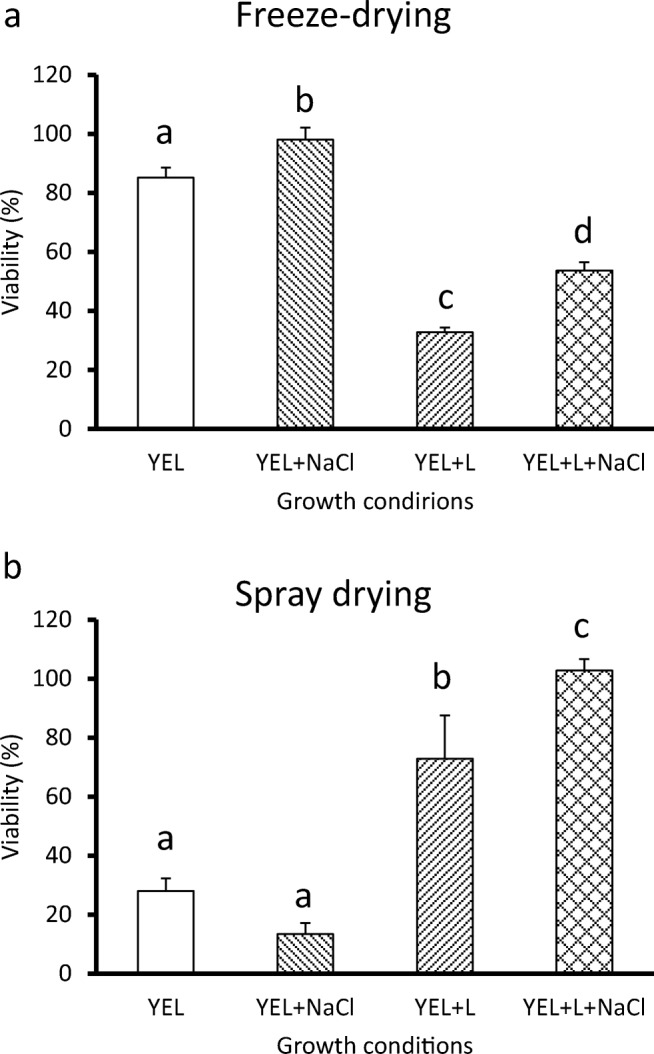
*P. freudenreichii* adaptation modulates *P. freudenreichii* viability during freeze-drying and spray-drying. *P. freudenreichii* CIRM-BIA 129 was cultivated until the beginning of the stationary phase in YEL medium, in the presence or absence of NaCl and of lactose. *P. freudenreichii* were harvested, and then suspended in a maltodextrin solution (20%). The suspension was freeze-dried (A) and spray-dried (B), as described in the “Materials and methods” section. Results are expressed as a percentage of survival. Error bars represent the standard deviation for triplicate experiments. Significant differences are reported with different letters above the columns (*p* > 0.05). YEL: yeast extract lactate. L: lactose

Different *P. freudenreichii* cultures, with or without the addition of salt, were subjected to freeze-drying or spray-drying in order to evaluate tolerance towards industrial drying processes. The presence of salt in YEL containing NaCl, and in YEL containing lactose and NaCl, had a significant positive impact on *P. freudenreichii* viability during freeze-drying (98.0% and 53.7%, respectively) compared to YEL and YEL containing lactose (85.2% and 32.8%, respectively) (Fig. 4(A)). The addition of lactose had a significant negative impact on *P. freudenreichii* viability during freeze-drying.

Concerning the spray-drying process, a similar significant positive effect of NaCl was observed for bacteria grown in YEL containing lactose and NaCl compared to YEL containing lactose (102% and 73%, respectively, Fig. 4(B)). In contrast, bacteria grown in YEL containing NaCl exhibited a significantly lower viability, compared to bacteria grown in YEL (13% and 28%, respectively, Fig. 4(B)).

### *P. freudenreichii* viability after spray-drying and powder storage

**Fig. 5:**
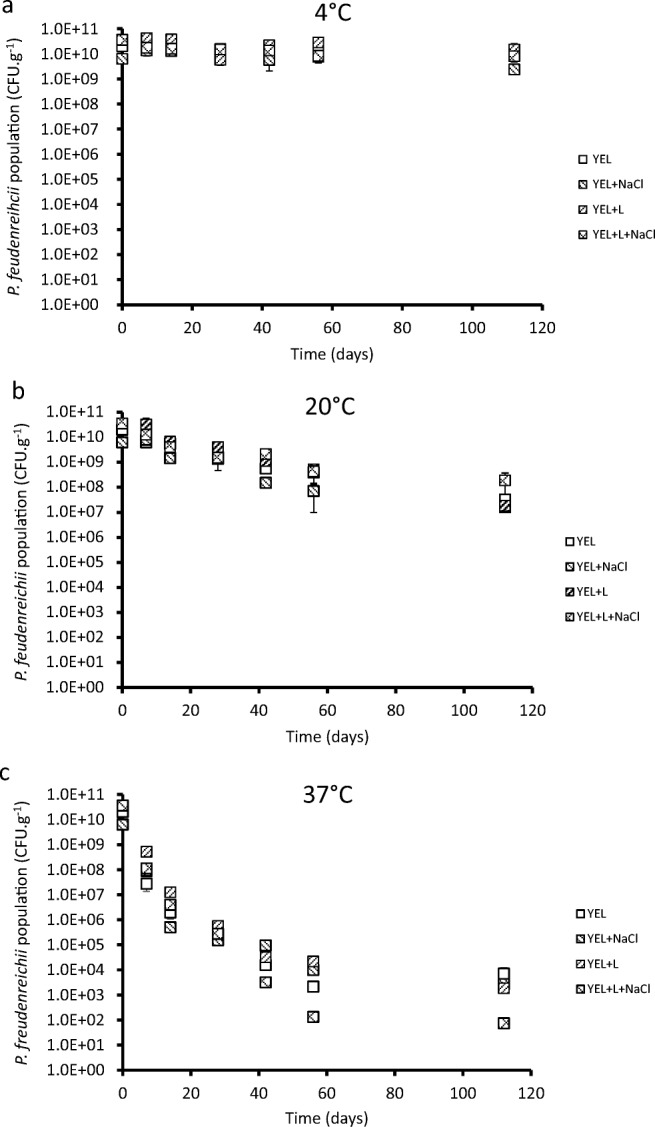
Storage temperature and *P. freudenreichii* adaptation modulates its viability during powder storage. The different powders were stored at 4 °C (**a**), 20 °C (**b**), and 37 °C (**c**) for 115 days. *P. freudenreichii* populations in spray-dried powders were quantified by CFU counting (as described in the “Material and methods” section) during storage. YEL: yeast extract lactate. L: lactose

The viability *P. freudenreichii* within spray-dried powders depends on growth conditions and on storage temperature. During storage at 4 °C, bacterial viability was stable over time (Fig. 5a). At 20 °C, the viability decreased. However, *P. freudenreichii* grown in YEL containing lactose and NaCl seemed to maintain a better viability over time, in comparison with the other growth conditions (Fig. 5b). During storage at 37 °C, *P. freudenreichii* viability decreased significantly (*p* < 0.05 at time 112 days) faster than during storage at 20 °C (Fig. 5b, c). At 37 °C, powders resulting from cultures in YEL medium containing lactose and NaCl exhibited a lower stability over time than the other cultures.

## Discussion

### The growth medium composition fine-tunes *P. freudenreichii* adaptation and osmoprotectants accumulation

This study revealed that *P. freudenreichii* is able to adapt to various concentrations of salt, and that different culture media support this adaptation. *P. freudenreichii* grew in the presence of 0.4 M NaCl, regardless of the medium. Growth was further observed up to 0.9 M NaCl in YEL medium and final populations were further enhanced by the addition of lactose. This indicates that, compared to milk ultrafiltrate (MU), YEL provides more potent osmoprotectants. It contains yeast extract and tryptone, which are sources of non-protein nitrogen (NPN) such as amino acids, peptides, glycine betaine, and soluble vitamins (Frings et al. 1993; Maria-Rosario et al. 1995; Robert et al. 2000). This large amount of NPN that provided potent osmoprotectants led to the efficient osmoadaptation of *P. freudenreichii* and a better tolerance of salt than MU and SW media, which contain less NPN. For all culture media, the salt concentration chosen was the highest that would allow *P. freudenreichii* to grow. However, in all salty growth media, *P. freudenreichii* exhibited slower growth compared to non-salty medium.

Previous studies clearly evidence accumulation of osmoprotectants as a result of osmotic stress, triggering osmoadaptation (Kets et al. 1996; Cardoso et al. 2007; Dalmasso et al. 2012; Huang et al. 2016c). The accumulated osmoprotectants were analyzed by NMR as described by Behrends et al. (2011), Pleitner et al. (2012), Vaidya et al. (2018), and Weinisch et al. (2019). During osmoadaptation, *P. freudenreichii* is able to accumulate glycine betaine, trehalose, and glutamate, as already reported (Huang et al. 2016c; Gaucher et al. 2019). Glycine betaine is an osmoprotectant which can be accumulated by numerous bacteria such as *P. freudenreichii*, *Lactococcus lactis*, and *L. plantarum* (Glaasker et al. 1996; Romeo et al. 2003; Huang et al. 2016c)*.* Accumulation of trehalose is a key constituent of stress response in *P. freudenreichii* (Cardoso et al. 2007; Dalmasso et al. 2012). More generally, this disaccharide is accumulated by different species in many stressful conditions such as acid, cold, osmotic, and oxidative stress (Cardoso et al. 2004, 2007; Thierry et al. 2011; Huang et al. 2016c). Glutamate accumulation in hyperosmotic conditions is well known and has been reported for *L. plantarum* (Kets et al. 1996; Glaasker et al. 1996). It constitutes the primary response to osmotic upshift and its transient accumulation is followed by its replacement with osmoprotectants such as trehalose in many types of cells (Csonka 1989).

**Fig. 6:**
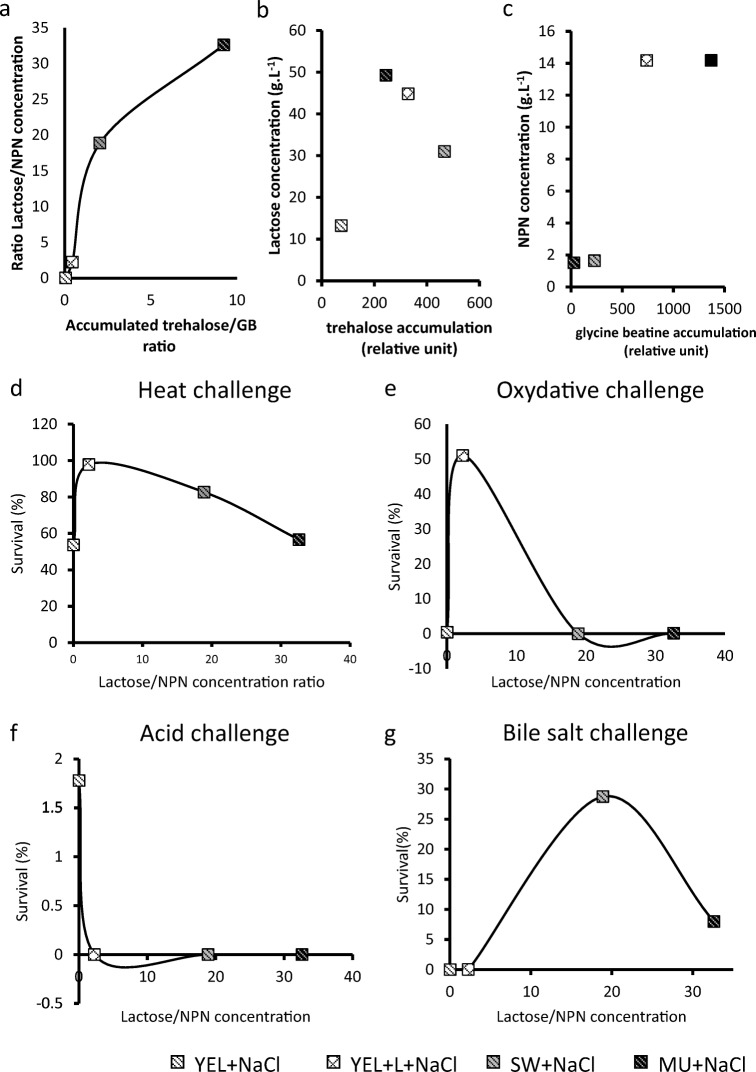
Fine-tuning the lactose/NPN ratio modulates physiological parameters including OP and stress tolerance. Data from the “Results” section were used to establish a correlation between the lactose/NPN ratio and the physiological parameters. The lactose/NPN ratio of growth media was plotted as a function of trehalose/NPN (A), the trehalose accumulation as a function of lactose concentration in growth media (B), and the GB accumulation as a function of NPN concentration in growth media. The lactose/NPN ratio was also plotted as a function of heat tolerance (D), of oxidative tolerance (E), of acid tolerance (F), and of bile salt tolerance (G). This suggests that the lactose/NPN ratio is correlated with the trehalose/GB ratio and that an optimal lactose/NPN ratio exists for each stress challenge

The YEL containing NaCl, which contains only lactate as a carbon source as well as large amounts of NPN, allowed the accumulation of high intracellular concentrations of GB. Other bacteria, including *Brevibacterium*, *Corynebacterium*, and *Enterococcus faecalis*, accumulate glycine betaine under hyperosmotic constraint in the presence of yeast extract (59, 66). The addition of lactose in YEL containing NaCl improved trehalose accumulation at the expense of GB accumulation. The same trend was observed in the dairy media. In MU, GB concentration is too low, whereas lactose is abundant (Table 1), leading to the accumulation of trehalose in hyperosmotic conditions. SW has a lower lactose/NPN ratio than MU (18.9 and 32.6, respectively), and *P. freudenreichii* accordingly accumulated osmoprotectants with a lower trehalose/glycine betaine ratio in SW containing NaCl than in MU containing NaCl (18.9 and 32.6, respectively). These data suggested a correlation between osmoprotectant accumulation and growth medium composition. We thus plotted the trehalose/GB ratio as a function of the lactose/NPN ratio (Fig. 6(A)), using values calculated from Table 1 and Fig. 2. This confirms such a correlation. In contrast, Fig. 6 (B) and (C) do not suggest any correlation between the lactose and intracellular trehalose provided per se or the NPN and intercellular GB provided per se. There is a correlation between the intracellular trehalose/GB ratio accumulated by *P. freudenreichii* and the lactose/NPN ratio of the growth medium composition (Fig. 6(A)). The higher the lactose/NPN concentration ratio is, the higher the trehalose/NPN ratio will be. Modulating the nitrogen and carbon composition of the growth medium thus made it possible to regulate the amount and composition of intercellular osmoprotectants in *P. freudenreichii.*

### Growth medium composition determines viability during lethal challenges

Accumulation of osmoprotectants may confer cross-protection towards various stress (Sheehan et al. 2006; Huang et al. 2016c, 2018). *P. freudenreichii*-osmoadapted cultures exhibited enhanced viability upon heat challenges, as already described (Huang et al. 2016c), regardless of the growth medium. This confirms that a cross-protection exists towards heat stress. *Escherichia coli* and *Pantoea agglomerans* also exhibited higher thermotolerance following GB accumulation favored by osmoadaptation (Teixido et al. 2005; Pleitner et al. 2012). In contrast, in our study, osmoadaptation decreased *P. freudenreichii* tolerance towards oxidative, acid, and bile salt challenges. Pichereau et al. (1999) reported a decreased bile salt tolerance in *Enterococcus faecalis* as a result of GB accumulation. In our study, the addition of lactose to YEL-type medium triggered high trehalose accumulation and increased *P. freudenreichii* tolerance towards heat, oxidative, acid, and bile salt challenges. Trehalose accumulation accordingly leads to enhanced tolerance of *L.s lactis* towards acid and bile salt challenges (Termont et al. 2006; Carvalho et al. 2011).

Huang et al. reported that *P. freudenreichii* osmoadaptation in hyperconcentrated SW, which contains high amounts of lactose, triggered increased tolerance towards heat, acid, and bile salt challenges. Hyperconcentrated SW imposed osmoadaptation to *P. freudenreichii*, without variation of the lactose/NPN ratio (Huang et al. 2018). This enhanced tolerance towards different challenges could thus be explained by trehalose accumulation during osmoadaptation and by the matrix protection of the hyperconcentrated SW.

We then plotted the survival rates upon the different stress challenges (from Fig. 3) as a function of the lactose/NPN ratio (from Table 1). For each challenge, the resulting curve (Fig. 6(D–G)) suggests that an optimal lactose/NPN ratio corresponds to an optimal survival rate. In these experiments, a ratio close to 2.3 correlated with the highest tolerance to heat and oxidative challenges (Fig. 6(B, C)), while a ratio close to 20 correlated with the highest tolerance to bile salt challenges (Fig. 6(D)). Acid tolerance of *P. freudenreichii* occurred when the accumulation of GB was the highest (Fig. 6(E)).

### Regulating *P. freudenreichii* osmoadaptation makes it possible to increase its survival during drying and storage

Different *P. freudenreichii* YEL cultures, with or without NaCl and with or without lactose, exhibited various survival rates upon drying. Concerning freeze-drying, osmoadaptation in YEL containing NaCl led to the best viability after freeze-drying. Such a protection seems to be correlated with the high accumulation of glycine betaine. Accumulation of GB can either increase *Lactobacillus salivarius* tolerance or decrease *Lactobacillus coryniformis* tolerance towards freeze-drying (Sheehan et al. 2006; Bergenholtz et al. 2012). Concerning spray-drying, the best tolerance was observed for *P. freudenreichii* grown in YEL containing lactose and NaCl. Its osmoadaptation in medium containing lactose, leading to more trehalose accumulation, allowed an enhanced adaptation towards spray-drying. This is in accordance with the enhanced tolerance towards heat and oxidative challenges (stresses encountered during spray-drying), which was observed in these cultures. Different osmoadaptations thus have different impacts on *P. freudenreichii* tolerance towards both drying processes. In contrast, GB accumulation in *L. salivarius* improved its resistance to freeze-drying and to spray-drying (Sheehan et al. 2006).

*P. freudenreichii* grown in YEL containing lactose and NaCl experienced a high viability loss during storage at high temperatures (Fig. 5c). *P. freudenreichii* adaptation can be used to obtain better viability during a particular process, but one culture condition cannot provide protection from all the technological stresses. To our knowledge, this is the first report showing that, in addition to triggering osmoadaptation, it is possible, in propionibacteria, to fine tune the trehalose/GB ratio and thus to modulate stress tolerance. Furthermore, this is the first report showing that fine-tuning trehalose accumulation and trehalose/GB ratio drastically increases survival upon spray-drying, a process known to induce massive cell death.

Propionibacteria adaptation can be used to optimize viability during specific industrial processes. Growth medium composition, like carbon sources, nitrogen sources, and the ratio thereof, can be controlled to optimize bacterial adaptation. However, an adaptation can improve bacterial viability during a technological stress but not during all the production and digestion processes.
